# Educational inequality in consumption of *in natura* or minimally processed foods and ultra-processed foods: The intersection between sex and race/skin color in Brazil

**DOI:** 10.3389/fnut.2022.1055532

**Published:** 2022-12-08

**Authors:** Barbara Virginia Caixeta Crepaldi, Letícia Martins Okada, Rafael Moreira Claro, Maria Laura da Costa Louzada, Leandro F. M. Rezende, Renata Bertazzi Levy, Catarina Machado Azeredo

**Affiliations:** ^1^Programa de Pós-Graduação em Ciências da Saúde, Faculdade de Medicina, Universidade Federal de Uberlândia, Uberlândia, MG, Brazil; ^2^Departamento de Nutrição, Escola de Enfermagem, Universidade Federal de Minas Gerais, Belo Horizonte, MG, Brazil; ^3^Departamento de Nutrição da Faculdade de Saúde Pública, Universidade de São Paulo, São Paulo, SP, Brazil; ^4^Departamento de Medicina Preventiva, Escola Paulista de Medicina, Universidade Federal de São Paulo, São Paulo, SP, Brazil; ^5^Departamento de Medicina Preventiva, Faculdade de Medicina, Universidade de São Paulo, São Paulo, SP, Brazil

**Keywords:** food consumption, *NOVA*, social inequalities, ultra-processed food, intersectionality

## Abstract

**Background:**

It remains uncertain how the intersection between educational, gender, and race/skin color inequalities influences food consumption in Brazil. In this study, we examined the educational inequality in the consumption of *in natura*/minimally processed and ultra-processed foods by Brazilians with an intersectional perspective between sex and race/color.

**Methods:**

We used cross-sectional data from the Telephone Surveillance System (VIGITEL 2019), comprising 52,443 participants ≥ 18 years. Daily food consumption was considered high when consumption of ≥5 foods for each food group was reported the day before the survey. Educational inequality in food consumption was assessed by the slope index of inequality (SII) and the relative index of inequality (RII) according to sex and race/color (White; Black/Brown). Positive SII and RII values > 1.0 indicate higher food consumption among more educated participants.

**Results:**

The consumptions of *in natura*/minimally processed and ultra-processed foods were more prevalent in those with the highest level of education (≥12 years) and intermediate education (9–11 years), respectively. However, highly educated White women had higher consumption of *in natura*/minimally processed foods than Black women with the same education level, and White men in low and intermediate school levels had higher consumption of these foods than Black men with the same education levels. We found higher absolute educational inequality for *in natura*/minimally processed foods among White women (SII 21.8, 95% CI 15.3, 28.4) and Black/Brown men (SII 19.3, 95% CI 12.5, 26.1). Black/Brown men (SII 7.3, 95% CI 0.5, 14.0) and Black/Brown women (SII 5.6, 95% CI 1.0, 10.2) had higher absolute educational inequality than White men (SII −3.3, 95% CI −10.9, 4.3; *P* = 0.04) in the consumption of ultra-processed foods.

**Conclusion:**

Educational inequalities influenced the consumption of *in natura*/minimally processed more than ultra-processed foods, and, for the latter, inequalities were greater among Black/Brown men and women than among White men.

## Introduction

The participation of ultra-processed foods in diet has grown in the last few decades in different countries, especially in low- and middle-income countries (1, 2). In Brazil, for example, *in natura* or minimally processed foods have being intensely replaced by ultra-processed foods (3, 4). Ultra-processed foods are formulations of many ingredients, mainly of industrial use exclusively, which contain little or no intact food in their composition. These products are often added with a series of additives that provide attractive sensory attributes, such as texture, smell, and/or taste (5). The increasing consumption of ultra-processed foods is problematic because results in a general deterioration in the dietary nutritional profile and increases the risk of developing non-communicable diseases (NCDs) (6, 7).

The socioeconomic level, assessed mainly by income and educational attainment, is one of the major determinants of populational dietary patterns. In low- and middle-income countries, higher educational attainment, and income have been associated both with healthier dietary patterns (with higher consumption of fruits and vegetables and whole grains), but also with a higher intake of ultra-processed foods, such as sweets and candies, concomitantly, and paradoxically (8–11). In Brazil, despite the paradoxical pattern, the consumption of some ultra-processed foods, such as instant noodles and reconstituted meat products, is already more frequent among socially disadvantaged individuals (12).

Concomitantly, other sociodemographic characteristics, such as sex and/or skin color, also exert a great influence on populational dietary patterns (9). Brown men and women have lower regular consumption of fruits and vegetables compared to their White counterparts, and Black/Brown men have a higher regular consumption of beans when compared to White men (13). However, these comparisons between sex-skin color groups have not properly considered differences in socioeconomic level (13, 14). Socioeconomic inequalities observed in Brazil may interact and overlap with the race/color and sex dimensions (15). There is limited understanding of how the intersectional nature of sex and skin color/race determines the magnitude of socioeconomic inequalities in food consumption of individuals. The perspectives of intersectionality consider that the dimensions of inequality, such as race/color and sex, are interact and interdependent, and that these are experienced simultaneously (16, 17). Social inequalities in food consumption should be considered for NCDs prevention and control in low- and middle-income countries (10). Monitoring of food consumption and the interaction of equity stratifiers (education, sex, and race/color) can contribute to the development of equitable health policies.

In this study, we aimed to identify and quantify the magnitude of educational inequalities in the consumption of *in natura*/minimally processed and ultra-processed foods among Brazilians in 2019, according to the intersection between sex and race/skin color.

## Methods

### Study population, sampling, and data source

We used data from participants of the Brazilian Surveillance of Risk and Protective Factors for Chronic Diseases through Telephone Interviews (VIGITEL 2019), adults 18 years or older, residing in households with a landline in the 26 Brazilian state capitals and the Federal District. VIGITEL is a cross-sectional monitoring system for the frequency and distribution of the main determinants of NCDs, managed by the Brazilian Ministry of Health (18).

The sampling process consisted of drawing telephone lines by city, stratified by zip code, followed by drawing an individual residing in a selected household. The sample was weighted in order to minimize possible sampling biases, considering the non-universal coverage of the fixed telephone system and the difference in the probability of each individual being selected for the study. A final weight was assigned to each respondent, considering the inverse of the number of telephone lines in the respondent’s household, the number of adults living in the household, and a third factor aiming to match the sociodemographic composition of the sample (sex, education, and age) in each city in 2019 to that of the entire population [based on official projection (Brazilian Institute of Geography and Statistics – IBGE] (Rake Method) (18). The final weight assigned to each interviewed participant (Rake Method), allows the correspondence of the population of individuals aged 18 years or more in each city, with and without fixed telephone line, in 2019 (18).

In the main analyses, we used data from the total sample (52,443 adults). The participants who did not know or did not want to inform their educational level (*n* 743; 1.4% of the initial sample) had their data imputed by VIGITEL, from the most frequently observed value considering sex and age (19). Information regarding self-declaration of race/skin color was audited for participants answering “others,” in order to standardize the classification of synonyms, especially frequent in the case of Brown individuals (people who declared themselves, for example, *morenos*). At the end of the audit, the Black and Brown categories were grouped.

In the subgroup analysis by sex and race/skin color, participants who declared themselves Yellow (*n* 427; 0.8% of the initial sample) and Indigenous (*n* 617; 1.2% of the initial sample) were excluded due to the low frequency, which limits the power to detect significant differences within the group. In these analyses, participants who did not know or did not want to declare their race/skin color (*n* 1.163; 2.2% of the initial sample) were also excluded, totaling 50,236 included participants.

### Study variables

#### Consumption of *in natura*/minimally processed and ultra-processed foods

Consumption of *in natura*/minimally processed (fruits, vegetables, roots and tubers, grains, legumes, meat, eggs, milk, nuts, and seeds) were evaluated in relation to the day before the interview, representing markers of healthy eating (18). In addition, consumption of ultra-processed foods (soft drinks, artificial juices, powdered drink mixes, powdered chocolate milk mix, flavored yogurt, salty snacks, sweet cookies and cake, sweet desserts, reconstituted meat products, bread, sauces, margarine, and ready-to-heat products) were evaluated in relation to the day before the interview, representing markers of unhealthy eating (18). The two food groups relied on the *NOVA* classification, which categorizes foods according to the extent and purpose of their processing (5). More details about the foods included in the *in natura*/minimally processed and ultra-processed food groups are provided in Supplementary material 1.

Food consumption was assessed through the following question: “Now I am going to list some foods and I would like you to tell me if you ate any of them yesterday (from when you woke up to when you went to sleep).” In order to assess the consumption of *in natura*/minimally processed and ultra-processed foods, respectively, the instruction was followed by questions in the following format: “I will start with *in natura* or staple foods [specific foods mentioned in the interview, described in Supplementary material 1] (yes or no),” and “Now I will list industrialized foods or products [specific foods mentioned in the interview, described in Supplementary material 1] (yes or no)” (18). The food questionnaire used by VIGITEL 2019 was based on instruments with satisfactory validity in Brazil, as documented by Sattamini (20).

A daily consumption equal to or higher than 5 different foods for each food group (*in natura*/minimally processed foods and ultra-processed foods) was considered as “high consumption,” in compliance with the assessment parameter adopted by VIGITEL (18). In the Brazilian context, a consumption equal to or greater than 5 groups of ultra-processed foods reflects a participation of about 44.0% of ultra-processed foods in the total caloric value of the diet (21).

#### Equity stratifiers

Food consumption was described according to years of schooling (0–3; 4–8; 9–11; ≥12 years) and the intersection between sex and race/skin color (White men; Black/Brown men; White women; Black/Brown women).

The categories of school level we used, are based on Brazilian cutoffs for illiterate/less than primary school (0–3 years of study), primary (elementary) school (4–8 years), secondary (middle) school (9–11 years) and higher education (≥12 years of study). In Brazil there is a marked difference between job types and income level based on these categories of education, thus we used these categories to capture not only the role of education, *per se*, but also indirectly the role of income. In addition to the use of complex measures of inequality that consider all levels of education, we aimed to verify the frequency of consumption of *in natura*/minimally processed foods and ultra-processed foods according to years of education. Considering that the different levels of education may present differences in food consumption, the use of ordered stratifiers with more than two categories is more informative.

### Statistical analysis

The sociodemographic characteristics and food consumption were expressed in means or relative frequencies for the total sample and subgroups with intersection between sex and race/color. For the presentation of inequalities in food consumption according to schooling in the total sample and subgroups, graphs of equiplot type were generated (Pelotas, Brazil).^1^

We calculated simple measures of inequality (22), such as absolute difference [most educated group (≥12 years of study) – least educated group (0–3 years of study)] and ratio (most educated group/least educated group) of the prevalence of consumption *in natura*/minimally processed and ultra-processed foods in the total sample and subgroups with intersection between sex and race/color.

### Complex measures of inequality

The magnitude of inequality in food consumption was estimated based on the level of education (educational inequality) through absolute (slope index of inequality – SII) and relative differences (relative index of inequality – RII) (22).

The SII represents the difference in the prevalence of food consumption between the more educated and less educated groups (difference in prevalence), whereas the RII represents the ratio of the prevalence between these groups. SII and RII consider all levels of schooling in the population instead of just comparing the two most extreme groups of schooling (23). SII and RII were estimated based on education for the total sample of participants and the subgroups with the intersection between sex and race/skin color.

Slope index of inequality and RII were estimated through logistic regression, a more appropriate analysis in the presence of prevalence indicators (24). The obtained SII values were multiplied by 100, ranging from −100 to +100, to facilitate visualization and understanding of results. Negative SII values indicate that food consumption is more prevalent in less educated groups, while positive values indicate a higher prevalence in more educated groups. SII values equal to ± 100 express total inequality, whereas zero represents a situation of total equality (absence of inequality) (22).

In general, the RII measures assume positive values (23), in which values farther from than 1.0 reflect higher levels of inequality (25). Results higher than 1.0 indicate a concentration of food consumption among more educated individuals and values lower than 1.0, including negative values, indicate a gradient in favor of the less educated ones (23, 25). If there is no inequality, the RII assumes a value of 1.0 (25).

SII and RII values were estimated with 95% confidence intervals. We performed the *t*-test to analyze whether educational inequality in food consumption assessed by SII and RII differed between subgroups (considering all possible comparisons). Associations with a value of *P* < 0.05 were considered statistically significant.

### Population attributable risk

In order to quantify inequality, we also calculated the population attributable risk, an absolute inequality measure that shows the possible improvement (in percentage points) in food consumption if all schooling categories had the same prevalence of food consumption as the most favored group (here considered ≥ 12 years of study) (22). The population attributable fraction, a relative inequality measure, represents the possible proportional improvement if there was no inequality between the categories of schooling. Higher results indicate more pronounced inequalities (22, 26).

Regarding population attributable risk, the prevalence of food consumption identified in the most favored group was subtracted from that in the total population (here considered for the total sample and the sample of each subgroup with intersection between sex and race/color). The population attributable fraction was obtained by dividing the absolute population attributable risk by the prevalence of food consumption in the total population (22).

Statistical analyses and graphs were performed using the Stata/SE software version 16 (StataCorp LLC, College Station, United States), considering the VIGITEL sample design for descriptive analyses (*Stata’s survey* module) and the weights of the sample in the estimation of SII and RII values.

## Results

In our study population, 54.0% correspond to women and 55.2% of the participants self-declared as Black/Brown race/color. The mean age among total sample was about 43 years old (SD 16.7) and the most frequent education category was between 9 and 11 years of study (38.4%). The sociodemographic distribution of participants according to the intersecting subgroups between sex and race/color and the percent of consumption of each food group is presented in Table 1. We identified a higher prevalence of participants with education between 9 and 11 years of study among Black/Brown men and women while the most frequent education category among White men and women was 12 or more years of study. In general, the percent of high consumption of *in natura*/minimally processed foods (≥5 different foods on the previous day) was low (29.8%), especially among Black/Brown men (25.2%), White men (29, 6%), and Black/Brown women (29.7%). The high consumption of ultra-processed foods (≥5 different foods on the previous day) was observed in almost a fifth of the total sample (18.2%), with a higher frequency among Black/Brown men (23.8%) and White men (20.0%) (Table 1).

**TABLE 1:** Sociodemographic characteristics, prevalence of consumption of *in natura*/minimally processed foods and ultra-processed foods in Brazil.^a^

General characteristics of the sample							Subgroups								
	Total sample			White men			Black/Brown men			White women			Black/Brown women		
				(38.7%)			(55.8%)			(42.9%)			(54.6%)		
					
	%		95% CI	%		95% CI	%		95% CI	%		95% CI	%		95% CI
Age (years)															
Mean		42.7			42.3			39.1			46.0			42.8	
SD		16.7			17.1			15.3			17.6			16.1	
Years of education															
0–3 years	06.3		05.9, 06.7	04.8		04.0, 05.7	06.1		05.2, 07.2	05.4		04.7, 06.2	07.2		06.5, 08.1
4–8 years	22.5		21.7, 23.4	18.5		16.7, 20.6	24.1		22.2, 26.1	20.8		19.4, 22.4	23.0		21.7, 24.4
9–11 years	38.4		37.5, 39.3	33.4		31.1, 35.8	45.3		43.2, 47.3	31.1		29.5, 32.8	42.0		40.4, 43.5
≥12 years	32.8		31.9, 33.7	43.2		40.8, 45.8	24.5		22.9, 26.3	42.6		40.8, 44.4	27.8		26.4, 29.2
High consumption of ^b^															
*In natura*/Minimally processed foods	29.8		28.9, 30.6	29.6		27.5, 31.8	25.2		23.5, 26.9	35.7		34.0, 37.4	29.7		28.3, 31.0
Ultra-processed foods	18.2		17.4, 19.0	20.0		18.0, 22.0	23.8		21.9, 25.7	14.8		13.3, 16.3	15.6		14.4, 16.9

VIGITEL, Surveillance of Risk and Protective Factors for Chronic Diseases through Telephone Interviews.

SD, standard deviation; CI, confidence interval.

^a^Sociodemographic characteristics, prevalence of consumption of each food group (*in natura/minimally* processed foods and ultra-processed foods), by intersection of sex and skin color/race, VIGITEL 2019.

^b^Consumption equal to or higher than 5 different foods from each food group, the day before the interview.

**FIGURE 1:**
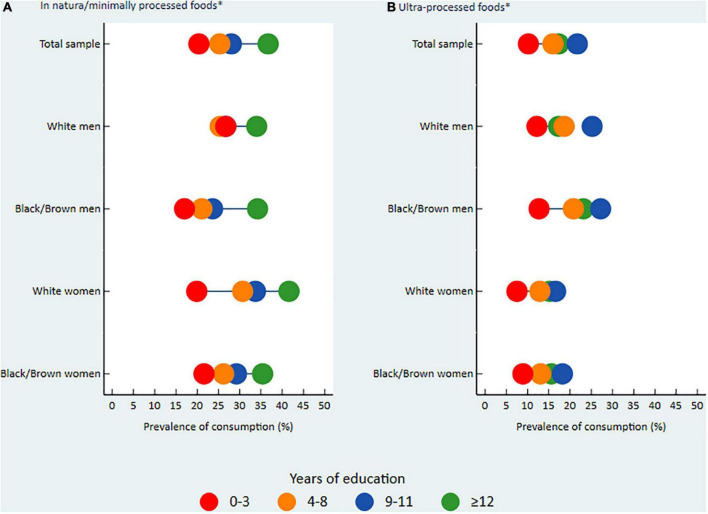
Prevalence of consumption of *in natura*/minimally processed foods **(A)** and ultra-processed foods **(B)** in Brazil.^a^ VIGITEL, Surveillance of Risk and Protective Factors for Chronic Diseases through Telephone Interviews. ^a^Prevalence of consumption of each food group [*in natura*/minimally processed foods **(A)** and ultra-processed foods **(B)**], by years of education and intersection of sex and skin color/race, VIGITEL 2019 (equiplot). *Consumption equal to or higher than 5 different foods from each food group, the day before the interview.

The consumption of *in natura*/minimally processed foods by the total sample and subgroups was lower among less-educated participants than the more educated ones. Although White men had a lower educational discrepancy, we observed an important educational gradient among White women (Figure 1A). At the highest level of education (≥12 years of study), White and Black/Brown men had a similar consumption frequency (approximately 34.0%). However, the prevalence of consumption was higher in less educated White men compared to Black/Brown men with intermediate education. Among more educated women, White women had a higher consumption frequency than Black/Brown women, especially when considering the highest level of education (41.6% vs. 35.4%). However, at the highest level of education, Black/Brown women had a consumption frequency similar to that of men (Black/Brown and White) (Figure 1A). In general, among the *in natura*/minimally processed foods included in this study, vegetables and nuts and seeds were the food categories that most clearly showed a pattern of consumption inequality favoring more educated individuals (see Supplementary material 2).

Differently, for the consumption of ultra-processed foods, a higher prevalence was identified among participants with intermediate education (9–11 years of study) in the total sample and subgroups. We observed a higher educational discrepancy between White and Black/Brown men. White men with intermediate education, and especially those with the highest level of education (≥12 years of study), had a lower frequency of consumption of ultra-processed foods compared to their respective Black/Brown counterparts (17.3% vs. 23.1% in the highest level of education). A slight educational discrepancy was observed between White and Black/Brown women (Figure 1B). The ultra-processed foods that were more frequently consumed by Brazilians were margarine and bread, with higher prevalence among participants with intermediate education and those with a higher level of education, respectively (see Supplementary material 3).

Among the most educated groups, the most frequent consumption of *in natura*/minimally processed foods and ultra-processed foods was also identified through positive SII results and RII results above 1.0 (Table 2). Educational inequality was higher for the consumption of *in natura*/minimally processed foods (SII 19.3, 95% CI 16.1, 22.5; RII 1.9, 95% CI 1.7, 2.1) than for ultra-processed foods (SII 3.6, 95% CI 0.7, 6.6; RII 1.2, 95% CI 1.0, 1.4), mainly identified by absolute measures. In general, the values found for the simple measures of inequality, difference, and ratio also confirmed that the consumption of these two food groups was more prevalent among more educated participants (Table 2).

**TABLE 2:** Consumption of *in natura*/minimally processed and ultra-processed foods, as well measures of inequality in Brazil.^a^

Consumption of food groups^b^	Simple measures of inequality				Complex measures of inequality				Population attributable risk (percentage points)	Population attributable fraction
				
Subgroups of sex and skin color/Race	% lowest education (0-3 years of study)	% highest education (≥ 12 years of study)	Absolute difference	Ratio	SII	95% CI	RII	95% CI		
*In natura*/Minimally processed foods										
Total sample	20.4	36.7	16.3	1.8	19.3	16.1, 22.5	1.9	1.7, 2.1	–6.9	–23.2
White men	26.7	34.0	7.3	1.3	14.7	5.8, 23.6	1.6	1.2, 2.1	–4.4	–14.9
Black/Brown men	17.0	34.2	17.2	2.0	19.3	12.5, 26.1	2.2	1.5, 2.8	–9.0	–35.7
White women	19.9	41.6	21.7	2.1	21.8	15.3, 28.4	1.9	1.5, 2.2	–5.9	–16.5
Black/Brown women	21.6	35.4	13.8	1.6	15.1	10.1, 20.1	1.7	1.4, 1.9	–5.7	–19.2
Ultra-processed foods										
Total sample	10.2	17.3	7.1	1.7	3.6	0.7, 6.6	1.2	1.0, 1.4	0.9	4.9
White men	12.2	17.3	5.1	1.4	–3.3	−10.9, 4.3	0.9	0.5, 1.2	2.7	13.5
Black/Brown men	12.7	23.1	10.4	1.8	7.3	0.5, 14.0*	1.4	1.0, 1,7	0.7	2.9
White women	07.5	15.2	7.7	2.0	4.7	−1.1, 10.5	1.4	0.8, 1.9	–0.4	–2.7
Black/Brown women	08.9	15.6	6.7	1.8	5.6	1.0, 10.2**	1.4	1.0, 1.8	0.0	0.0

VIGITEL, Surveillance of Risk and Protective Factors for Chronic Diseases through Telephone Interviews.

SII, slope of inequality index; CI, confidence interval; RII, relative inequality index.

Difference = Highest education − Lowest education; Ratio = Highest education/Lowest education.

SII and RII values were significantly different according to *t*-test.

^a^Prevalence of consumption of each food group (*in natura*/minimally processed foods and ultra-processed foods), as well as simple and complex measures of inequality and absolute and relative population attributable risk by intersection of sex and skin color/race, VIGITEL 2019.

^b^Consumption equal to or higher than 5 different foods from each food group, the day before the interview.

*Difference between White men and Black/Brown men (*P*-value = 0.04).

**Difference between White men and Black/Brown women (*P*-value = 0.04).

The consumption of *in natura*/minimally processed foods remained concentrated among the most educated individuals in all intersectional subgroups of sex and race/skin color. The magnitude of absolute and relative inequalities was higher in Black/Brown men (SII 19.3, 95% CI 12.5, 26.1; RII 2.2, 95% CI 1.5, 2.8) and White women (SII 21.8, 95% CI 15.3, 28.4; RII 1.9, 95% CI 1.5, 2.2). The population attributable risk results indicate that, if educational inequality were eliminated, the consumption of *in natura*/minimally processed foods would increase by 9.0 percentage points and 35.7% for Black/Brown men and 5.7 percentage points and 19.2% among Black/Brown women (Table 2).

In the analysis of the magnitude of educational inequality in the consumption of ultra-processed foods, we identified a significant absolute inequality for Black/Brown men (SII 7.3, 95% CI 0.5, 14.0) and Black/Brown women (SII 5.6, 95% CI 1.0, 10.2), but not for White men and women. However, we observed among White men a possibly higher consumption of ultra-processed foods in the least educated individuals (SII −3.3, 95% CI −10.9, 4.3; RII 0.9, 95% CI 0.5, 1.2). In the comparison between subgroups, Black/Brown men and women showed a higher absolute inequality in relation to White men (*P* = 0.04, for both). If educational inequality ceased to exist among White men, there would be a reduction of 2.7 percentage points and 13.5% in the consumption of ultra-processed foods (Table 2).

## Discussion

The present study showed that the consumption of *in natura*/minimally processed foods was more prevalent in participants with a higher level of education, while that of ultra-processed foods was more frequent among those with intermediate education in the total sample and all analyzed subgroups with the intersection between sex and race/skin color. In middle-income countries, such as Brazil, the level of education influences the employability and income of the population (27, 28). Higher educational level impacts on a higher socioeconomic level (29), and influences healthy food choices even in the lower income population (30). In addition, considering that the price of healthy foods, such as fruits and vegetables, tends to be higher than the price of ultra-processed foods in Brazil (31), such factors may justify the more frequent consumption of *in natura*/minimally processed foods among the most educated in the present study. On the other hand, the more frequent consumption of ultra-processed foods among people with intermediate education (9–11 years of study) is possibly due to the considerable participation of this group in the informal labor market, characterized by intense working (32) that can make it difficult to buy and prepare food/drinks at home (33).

The consumption of *in natura*/minimally processed foods was the food group that showed the highest absolute educational inequality, especially between White women and Black/Brown men, while White men showed lower absolute and relative inequality. Additionally, in the consumption of ultra-processed food, Black/Brown men and women had a significantly higher absolute inequality than White men. Elimination of educational inequality would result in increased consumption of *in natura*/minimally processed foods of 36.0% for Black/Brown men and 19.0% for Black/Brown women. In addition, the elimination of educational inequality among White men would reduce the consumption of ultra-processed food by 13.5% in this group.

We identified that Black/Brown men with low to intermediate levels of education consumed less *in natura*/minimally processed foods than White men with lower levels of education. Considering that a higher level of education implies better wages in Brazil (27) and that having higher incomes increases the opportunity for adherence to the consumption of healthy foods (34), it would be expected that the intermediate education level of Black/Brown men would favor higher access to and consumption of these foods in relation to less educated White men. However, in a country marked by a historical and expressive racial inequality in social indicators, Black/Brown men only had the same prevalence of healthy food consumption as White men in the highest level of education. This result may be explained by the fact that, although the unemployment rate is higher among Black/Brown people than among White people, regardless of education, this difference is relatively smaller among those with a higher level of education, although the wage disadvantages remain expressive (27).

We identified that Black/Brown women with the highest level of education achieved the prevalence of consumption of *in natura*/minimally processed foods of their respective counterparts of White men and Black/Brown men, despite Black/Brown women having the worst work income in Brazil (for example, 44.4% of White men’s earnings) (35). Unequally, considering that education determined more the differences in the consumption of healthy foods among White women than among the other subgroups, we observed, in the highest level of education, an expressive lower frequency of consumption of healthy foods among Black/Brown women in relation to White women. Brazilian women have better educational indicators compared to men, and White women have higher work incomes than Black/Brown women (35). The greater education of women associated with higher income among White women may have contributed to the higher consumption of healthy foods in this group (11, 28, 36). Healthy eating patterns among Brazilian women, especially those with higher education and income, have been identified in other epidemiological studies (8, 9, 37).

We also identified an important privilege of White skin among more educated men, favoring a lower frequency of consumption of ultra-processed foods in relation to their Black/Brown male counterparts. While higher levels of education seem to facilitate the consumption of ultra-processed products among Black/Brown men and women, educational inequality among White men showed that the consumption of ultra-processed food already seems to reach the less socioeconomically advantaged population in a more concentrated manner. The White men’s wage advantages in relation to all subgroups (35) may have contributed to this finding.

The consumption of ultra-processed foods reaching White men with less education in a more concentrated manner and a slighter educational discrepancy in the subgroups for this food group reinforce the occurrence of the food transition process in Brazil (9), which has already occurred in developed countries (38, 39). The progression of the dietary pattern transition is characterized by higher consumption of cheaper, more caloric, and less nutritious foods at lower educational and income levels (1, 38, 39). In developing countries, such as Brazil, unhealthy food options are increasingly more accessible (40). On the other hand, healthy foods such as fruits and vegetables observed continuous increases in its price (40).

Projections of the price of healthy and unhealthy foods from 2017 to 2030 in Brazil, using data from 1995 to 2017, showed that the price difference between these foods reduced over time and forecasted that ultra-processed foods will become cheaper than *in natura*/minimally processed foods as of 2026 (41). This would increase barriers to the consumption of healthy foods (42) and encourage the consumption of unhealthy foods, specially by people with lower incomes, as a way to control expenditures (34), widening dietary and health inequalities. The growing participation of ultra-processed foods in the diet of the Brazilian population has been observed in recent decades (3), including in the most vulnerable populations (12). Data from the Brazilian Household Budget Surveys 2017-2018, show that ultra-processed foods already contribute about one-fifth (19.7%) of the calories consumed by Brazilians, and that the consumption of some ultra-processed foods, such as instant noodles and reconstituted meat products, is more frequent in the lowest income quartiles of the population (12). In addition to the economic factor, food consumption can be influenced by other issues such as cultural, ethnic, and perceived racial discrimination (14, 42).

Robust epidemiological evidence on negative health effects associated with the consumption of ultra-processed foods has been described (6). High consumption of ultra-processed foods has been associated with higher calorie intake, body fat gain (43), overweight/obesity risk (6), type-2 diabetes (44), breast cancer (45), cardiovascular diseases, depression, and mortality for all the causes (6). Specifically for obesity, a study in the North American population showed that, for all levels of education, the age-adjusted prevalence of overweight/obesity was 44.0% higher in Black women than in White women and 2.0% higher in Black men than in White men (46). Thus, the consumption of ultra-processed foods may increase overweight, especially in the Black population, with emphasis on women, accentuating health inequalities.

It is expected that the consumption of ultra-processed foods will become more frequent in more vulnerable populations within a few years, such as Black/Brown and less educated individuals, if intersectoral and effective interventions are not articulated and adopted quickly. The guidelines for promoting the consumption of healthy foods and discouraging the consumption of unhealthy foods must be accompanied by plausible cultural and economic strategies for population adherence, especially the less educated and poorer (34, 42), because the ongoing COVID-19 pandemic enhances social, racial, and gender inequalities that already exist in Brazil (47). Public policies aimed at subsidizing *in natura* or minimally processed foods (48), may be effective in increasing purchase and consumption power of these foods by the vulnerable population (48, 49). In addition, a favorable a food environment with a high density of healthy eating establishments, such as street markets, is needed (50).

In addition to subsidizing healthy foods, the taxation of ultra-processed foods may discourage their consumption, especially by the most vulnerable population (48, 49). In Brazil, the price of ultra-processed foods was inversely associated with the prevalence of overweight and obesity, especially protecting individuals with higher socioeconomic vulnerability (51). In the same way, food marketing regulation can also contribute to improving the quality of the population’s diet (49). A new labeling system was recently approved in Brazil, encompassing frontal nutrition labeling and a magnifying glass design to identify high nutrient content, including added sugars (52). Despite this, the Brazilian model differs from those adopted by other Latin American countries and jurisdictions in the United States, with scientific evidence of beneficial effects (53). Complementary measures are needed to reduce the persuasive advertising of ultra-processed foods on television and the internet, identified in Latin American countries, including Brazil (54–56).

Our results show the importance of ensuring access to better educational opportunities, with emphasis on the Black/Brown population, as a way to improve the quality of food consumption. It is noteworthy that, despite the growing participation of the Black/Brown population in higher education in recent years, due to the increase in the number of places and the policies of democratization of access to public higher education, the percentage of Black/Brown individuals with complete higher education is still low compared to White population (32.0% *vs.* 66.0% in 2017) (57, 58). In addition to the benefit of education in the employability and income of the population (27), knowledge, including specific to health (28), such as information on types and characteristics of foods that contribute to making them more or less healthy, may also influence an adequate diet (42).

Our study has some limitations. VIGITEL collected data from individuals with access to a landline residing in Brazilian state capitals and the Federal District. Although weighting factors have been used to mitigate this limitation, the inclusion of the population without a landline in the System (having only a cell phone) has the potential to impact the estimates, such as a higher frequency of consumption of some ultra-processed foods (59). There is a possibility that respondents have a higher socioeconomic level than the population of capitals in general, because, in Brazil, access to fixed telephone lines has been reduced in recent years (60), and more socioeconomically privileged families are more likely to have a landline (61). Nevertheless, we identified the presence of expressive educational inequalities with the intersection between sex and race/color in food consumption. We did not include participants who declared themselves either Yellow or Indigenous in the intersection analysis due to limited sample size. This study innovates and advances in the perspective of intersectionality, showing the overlapping of inequalities in food consumption in Brazil. Another limitation is that the self-reported information made available by VIGITEL is subject to misclassification. However, there is an indication of good reproducibility and adequate validity of indicators of food and beverage consumption obtained through telephone surveys (62). Although VIGITEL used a method based on a list of foods (simplified food questionnaire) and not the open 24-h dietary recall, the simplified version was based on instruments with satisfactory validity, and the lack of quantification of consumption did not impact the understanding of the quality of the diet in the healthy and unhealthy dimensions proposed by the instrument (20, 21).

The strengths of the study are the analysis of the magnitude of social inequality in the consumption of two food groups (*in natura*/minimally processed and ultra-processed foods) of the *NOVA* classification, which has become a world reference for effective assessment of the quality of diets and their consequences in all forms of malnutrition (1, 4, 5). We used complex measures of inequality that took into account all levels of education of the representative sample of the population of the state capitals and the Federal District of an upper-middle-income country (UMIC) (63). Furthermore, our study fills a gap in the literature on the role of the intersectionality of sex and race/skin color in food consumption.

The present study has important implications for public health as it identifies the magnitude with which social inequality presents itself and the groups that are the most vulnerable to the consumption of foods classified according to the degree of processing. Our results can subsidize policies that equitably guarantee the possibility of adopting the golden rule: “always prefer *in natura* or minimally processed foods and freshly made dishes and meals to ultra-processed foods” presented in the renowned Dietary Guidelines for the Brazilian Population, which has a language accessible to less educated people (42). A variety in the consumption of *in natura* and minimally processed foods and the reduction in the consumption of ultra-processed foods goes beyond the fundamental importance of promoting healthy eating for the population (20). It implies the promotion of an environmentally sustainable and socially fairer food system (42, 64), mitigating the deepening of social inequalities caused by the food system that produces ultra-processed products (42).

We conclude that the inequality in the consumption of *in natura*/minimally processed foods favors more educated individuals. The prevalence of consumption of ultra-processed foods is more concentrated among those with intermediate education. Educational inequalities influence more the consumption of healthy than unhealthy foods. Among the subgroups, Black/Brown men with intermediate education had lower consumption of *in natura*/minimally processed foods when compared to White men with lower education level, showing the impact of racism on diet. Absolute inequalities for the consumption of unhealthy foods presented a higher magnitude between Black/Brown men and women when compared to White men, which may drive inequalities in NCD. The increase in education could impact an expressive increase in the consumption of healthy foods in the Black/Brown population, and in the reduction in the consumption of unhealthy foods in White men, but without changes in Black/Brown women.

## Data availability statement

The original contributions presented in this study are included in this article/Supplementary material, further inquiries can be directed to the corresponding author.

## Ethics statement

The studies involving human participants were reviewed and approved by the National Research Ethics Commission (CONEP) of the National Health Council/Brazilian Ministry of Health: Certificate of Presentation and Ethical Appreciation (CAAE) number 65610017.1.0000.0008. The patients/participants provided their written informed consent to participate in this study.

## Author contributions

BC and CA participated in the conception and design of the study, statistical analyses, data interpretation, and manuscript writing. RC and LO participated in the statistical analyses, data interpretation, manuscript writing, and review. LR, RL, RC, and ML participated in the interpretation of data, manuscript writing, and review. All authors carried out a critical review of the manuscript’s important intellectual content, approved the submitted final version, agreed to be responsible for all aspects of the work, and ensuring that issues related to the accuracy or integrity of any part of the work are investigated and resolved appropriately.

## Footnotes

1
www.equidade.org/equiplot
