# Supplementary material for: Educational inequality in consumption of in natura or minimally processed foods and ultra-processed foods: The intersection between sex and race/skin color in Brazil
Source: Front Nutr. 2022 Dec 8;9:1055532. doi: 10.3389/fnut.2022.1055532 (PMC9774479; doi:10.3389/fnut.2022.1055532)
Supplement: Supplementary file 1 [file Data_Sheet_1.PDF]

**Additional file 1:** Description of the foods included in each food group (*in natura*/minimally processed foods and ultra-processed foods) analyzed in the present study, VIGITEL 2019.

| Group | Foods | Specific foods mentioned in the interview |
|---|---|---|
| *In natura*/ minimally processed foods | Fruits (group 1) | Papaya, mango, melon, or pequi |
| | Fruits (group 2) | Orange, banana, apple, or pineapple |
| | Vegetables (group 1) | Lettuce, kale, broccoli, watercress, or spinach |
| | Vegetables (group 2) | Pumpkin, carrots, sweet potatoes, or okra/caruru |
| | Vegetables (group 3) | Tomato, cucumber, zucchini, eggplant, chayote, or beetroot |
| | Roots and tubers | Potato, cassava, or yam |
| | Grains (cereal) | Rice, noodles, polenta, couscous, or sweet corn |
| | Legumes | Beans, pea, lentils, or chickpea |
| | Meat | Beef, pork, chicken, or fish |
| | Eggs | Fried, boiled, or scrambled eggs |
| | Milk | Milk |
| | Nuts and seeds | Peanut, cashew nut, or Brazilian nut |
| Ultra-processed foods | Soft drink | Soft drink |
| | Artificial juices | Fruit juice in box or can |
| | Powdered drink mixes | Powdered drink mixes |
| | Powdered chocolate milk mix | Powdered chocolate milk mix |
| | Flavored yogurt | Flavored yogurt |
| | Salty snacks | Potato chips or salty biscuits |
| | Sweet cookies and cakes | Sweet cookie, sandwich cookie, or industrialized cakes |
| | Sweet deserts | Chocolate, ice cream, jelly, flan, or other industrialized desserts |
| | Reconstituted meat products | Hot dog, sausage, mortadella, or ham |
| | Bread | Sliced, hot dog, or hamburger bread |
| | Sauces | Mayonnaise, catchup, or mustard |
| | Margarine | Margarine |
| | Ready-to-heat products | Instant noodles, packet soup, frozen lasagna, or other ready-to-eat dish purchased frozen |

VIGITEL, Surveillance of Risk and Protective Factors for Chronic Diseases through Telephone Interviews.

**Additional file 2:** Prevalence of consumption of *in natura*/minimally processed foods in Brazil, by years of education, sex, and skin color/race, VIGITEL 2019.

| **Subgroups** | ***In natura*/minimally processed foods**[a] **Food category** | | | | | | | | | | | | | | | | | | | | | | | |
|---|---|---|---|---|---|---|---|---|---|---|---|---|---|---|---|---|---|---|---|---|---|---|---|---|
| Years of education, % | Fruits (group 1) | | Fruits (group 2) | | Vegetables (group 1) | | Vegetables (group 2) | | Vegetables (group 3) | | Roots and tubers | | Grains (cereal) | | Legumes | | Meat | | Eggs | | Milk | | Nuts and seeds | |
| | % | 95% CI | % | 95% CI | % | 95% CI | % | 95% CI | % | 95% CI | % | 95% CI | % | 95% CI | % | 95% CI | % | 95% CI | % | 95% CI | % | 95% CI | % | 95% CI |
| Total sample | | | | | | | | | | | | | | | | | | | | | | | | |
| Total | 34.4 | 33.5, 35.2 | 71.1 | 70.2, 72.0 | 50.9 | 50.0, 51.9 | 46.1 | 45.2; 47.0 | 67.6 | 66.7; 68.5 | 35.9 | 35.0; 36.8 | 84.9 | 84.1; 85.5 | 71.6 | 70.8; 72.5 | 88.5 | 87.8; 89.1 | 44.3 | 43.3; 45.2 | 56.5 | 55.5; 57.4 | 16.6 | 15.9; 17.3 |
| 0-3 years | 35.2 | 31.9, 38.7 | 66.1 | 62.7, 69.3 | 37.8 | 34.5, 41.2 | 39.7 | 36.4; 43.0 | 55.5 | 52.0; 58.8 | 31.4 | 28.1; 34.9 | 82.5 | 79.9; 84.9 | 71.7 | 68.7; 74.6 | 85.6 | 83.2; 87.7 | 42.2 | 38.7; 45.7 | 62.2 | 58.8; 65.5 | 11.0 | 08.9; 13.5 |
| 4-8 years | 35.0 | 33.1, 36.9 | 71.6 | 69.6, 73.5 | 43.6 | 41.5, 45.7 | 42.9 | 40.8; 44.9 | 62.7 | 60.6; 64.7 | 33.6 | 31.6; 35.6 | 84.4 | 82.7; 85.9 | 72.9 | 71.1; 74.7 | 86.0 | 84.3; 87.6 | 43.3 | 41.2; 45.5 | 58.9 | 56.8; 60.9 | 12.4 | 11.1; 13.9 |
| 9-11 years | 32.2 | 30.9, 33.5 | 69.7 | 68.2, 71.1 | 48.0 | 46.5, 49.5 | 45.7 | 44.2; 47.2 | 66.5 | 65.0; 67.9 | 36.1 | 34.7; 37.6 | 87.2 | 86.1; 88.2 | 74.1 | 72.8; 75.3 | 88.1 | 87.0; 89.1 | 44.9 | 43.4; 46.4 | 55.9 | 54.4; 57.4 | 14.5 | 13.6; 15.6 |
| ≥ 12 years | 36.3 | 34.8, 37.8 | 73.4 | 71.9, 74.8 | 61.8 | 60.2, 63.4 | 50.0 | 48.4; 51.7 | 74.6 | 73.1; 76.0 | 38.1 | 36.5; 39.7 | 82.9 | 81.6; 84.2 | 67.9 | 66.3; 69.4 | 91.1 | 90.0; 92.1 | 44.5 | 42.9; 46.2 | 54.4 | 52.7; 56.1 | 22.9 | 21.6; 24.3 |
| Men | | | | | | | | | | | | | | | | | | | | | | | | |
| Total | 28.9 | 27.6, 30.2 | 68.9 | 67.4, 70.4 | 47.8 | 46.3; 49.4 | 42.5 | 41.0; 44.0 | 66.1 | 64.6; 67.6 | 36.7 | 35.2; 38.2 | 87.7 | 86.6; 88.7 | 76.9 | 75.7; 78.2 | 88.7 | 87.5; 89.7 | 45.5 | 44.0; 47.0 | 54.1 | 52.5; 55.6 | 15.6 | 14.6; 16.7 |
| 0-3 years | 33.0 | 27.5, 39.0 | 64.4 | 58.6, 69.9 | 37.0 | 31.5; 42.9 | 36.2 | 30.8; 41.9 | 54.0 | 48.1; 59.8 | 32.6 | 26.9; 38.8 | 88.0 | 84.5; 90.8 | 79.8 | 75.6; 83.5 | 87.1 | 83.5; 90.0 | 45.6 | 39.7; 51.7 | 54.7 | 48.7; 60.5 | 10.7 | 07.4; 15.2 |
| 4-8 years | 28.8 | 25.9, 31.9 | 68.5 | 65.1, 71.7 | 39.3 | 35.9; 42.8 | 39.2 | 35.9; 42.6 | 61.8 | 58.2; 65.2 | 34.1 | 30.8; 37.5 | 86.5 | 83.7; 88.9 | 78.6 | 75.6; 81.3 | 84.3 | 81.1; 87.0 | 46.0 | 42.5; 49.6 | 55.0 | 51.4; 58.4 | 11.9 | 09.6; 14.5 |
| 9-11 years | 27.8 | 25.8, 29.9 | 67.3 | 65.0, 69.6 | 44.5 | 42.2; 46.9 | 42.2 | 40.0; 44.6 | 64.2 | 61.8; 66.6 | 36.4 | 34.1; 38.7 | 89.7 | 88.2; 91.0 | 79.0 | 77.1; 80.7 | 88.4 | 86.7; 90.0 | 46.2 | 43.8; 48.5 | 53.8 | 51.4; 56.1 | 13.8 | 12.4; 15.4 |
| ≥ 12 years | 29.6 | 27.2, 32.0 | 72.1 | 69.6, 74.4 | 60.3 | 57.6; 62.9 | 46.5 | 43.9; 49.2 | 74.1 | 71.7; 76.4 | 39.7 | 37.1; 42.4 | 85.9 | 83.8; 87.7 | 72.6 | 70.0; 74.9 | 92.4 | 90.9; 93.8 | 44.3 | 41.6; 47.0 | 53.7 | 51.0; 56.4 | 21.7 | 19.8; 23.8 |
| Women | | | | | | | | | | | | | | | | | | | | | | | | |
| Total | 39.0 | 37.9, 40.1 | 73.0 | 71.9, 74.0 | 53.6 | 52.4, 54.7 | 49.2 | 48.0; 50.3 | 68.8 | 67.7; 69.9 | 35.3 | 34.2; 36.4 | 82.4 | 81.5; 83.4 | 67.1 | 66.0; 68.2 | 88.3 | 87.4; 89.1 | 43.2 | 42.1; 44.4 | 58.5 | 57.4; 59.7 | 17.4 | 16.6; 18.3 |
| 0-3 years | 36.9 | 33.1, 41.0 | 67.3 | 63.2, 71.1 | 38.4 | 34.5, 42.6 | 42.4 | 38.4; 46.4 | 56.6 | 52.6; 60.5 | 30.4 | 26.7; 34.4 | 78.4 | 74.5; 81.8 | 65.5 | 61.4; 69.4 | 84.5 | 81.2; 87.3 | 39.5 | 35.4; 43.7 | 68.0 | 64.2; 71.6 | 11.2 | 08.8; 14.2 |
| 4-8 years | 40.5 | 38.2, 42.9 | 74.4 | 72.1, 76.5 | 47.3 | 44.8, 49.8 | 46.1 | 43.6; 48.5 | 63.5 | 61.1; 65.8 | 33.2 | 31.0; 35.5 | 82.4 | 80.4; 84.3 | 67.9 | 65.6; 70.2 | 87.5 | 85.7; 89.1 | 41.0 | 38.6; 43.4 | 62.3 | 59.9; 64.7 | 12.9 | 11.5; 14.4 |
| 9-11 years | 36.2 | 34.5, 37.9 | 71.8 | 70.0, 73.6 | 51.2 | 49.3, 53.1 | 48.9 | 47.0; 50.8 | 68.5 | 66.7; 70.3 | 35.9 | 34.1; 37.7 | 84.8 | 83.3; 86.2 | 69.6 | 67.9; 71.3 | 87.8 | 86.3; 89.2 | 43.8 | 41.9; 45.6 | 57.9 | 56.0; 59.8 | 15.2 | 14.0; 16.6 |
| ≥ 12 years | 41.5 | 39.5, 43.5 | 74.4 | 72.5, 76.2 | 63.1 | 61.1, 65.0 | 52.7 | 50.6; 54.8 | 74.9 | 73.1; 76.6 | 36.8 | 34.9; 38.7 | 80.6 | 78.8; 82.4 | 64.2 | 62.2; 66.2 | 90.0 | 88.6; 91.4 | 44.8 | 42.7; 46.8 | 55.0 | 52.9; 57.0 | 23.9 | 22.2; 25.6 |
| White | | | | | | | | | | | | | | | | | | | | | | | | |
| Total | 35.8 | 34.4, 37.2 | 73.2 | 71.8, 74.5 | 57.5 | 56.0, 59.0 | 46.4 | 44.9; 47.8 | 69.7 | 68.3; 71.1 | 36.5 | 35.1; 37.9 | 83.1 | 81.9; 84.3 | 68.7 | 67.4; 70.1 | 88.8 | 87.7; 89.7 | 43.6 | 42.1; 45.1 | 57.0 | 55.6; 58.5 | 18.9 | 17.8; 20.0 |
| 0-3 years | 38.6 | 33.2, 44.2 | 66.6 | 61.2, 71.5 | 41.0 | 35.8, 46.5 | 36.9 | 31.9; 42.1 | 53.7 | 48.3; 59.1 | 30.3 | 25.3; 35.9 | 82.3 | 78.1; 85.8 | 70.4 | 65.4; 75.0 | 85.9 | 82.3; 88.9 | 41.7 | 36.4; 47.3 | 62.1 | 56.7; 67.2 | 10.5 | 07.3; 14.8 |
| 4-8 years | 37.6 | 34.6, 40.8 | 74.4 | 71.3, 77.3 | 48.6 | 45.3, 51.9 | 43.6 | 40.4; 46.9 | 65.1 | 61.9; 68.2 | 33.4 | 30.4; 36.6 | 83.7 | 80.9; 86.1 | 71.8 | 68.8; 74.5 | 85.3 | 82.7; 87.6 | 43.0 | 39.7; 46.3 | 64.0 | 60.8; 67.1 | 12.9 | 10.9; 15.3 |
| 9-11 years | 31.5 | 29.4, 33.6 | 72.3 | 69.7, 74.7 | 53.9 | 51.3, 56.4 | 46.0 | 43.5; 48.5 | 66.6 | 64.0; 69.1 | 37.5 | 35.1; 39.9 | 86.5 | 84.6; 88.2 | 72.9 | 70.8; 75.0 | 88.4 | 86.6; 90.1 | 43.7 | 41.2; 46.2 | 55.3 | 52.7; 57.8 | 15.9 | 14.3; 17.6 |
| ≥ 12 years | 37.8 | 35.6, 40.1 | 74.0 | 71.9, 76.0 | 66.3 | 64.0, 68.5 | 49.1 | 46.7; 51.4 | 76.1 | 74.1; 78.0 | 37.9 | 35.6; 40.2 | 80.4 | 78.4; 82.3 | 64.0 | 61.6; 66.2 | 90.9 | 89.4; 92.3 | 43.9 | 41.6; 46.3 | 54.5 | 52.2; 56.9 | 24.8 | 23.0; 26.8 |
| Black/Brown | | | | | | | | | | | | | | | | | | | | | | | | |
| Total | 33.0 | 31.9, 34.2 | 70.0 | 68.8, 71.2 | 46.3 | 45.0, 47.6 | 46.0 | 44.8; 47.3 | 66.2 | 64.9; 67.4 | 35.6 | 34.4; 36.8 | 86.1 | 85.2; 87.0 | 73.8 | 72.7; 74.9 | 88.4 | 87.4; 89.3 | 44.6 | 43.3; 45.9 | 56.2 | 54.9; 57.5 | 15.1 | 14.3; 16.0 |
| 0-3 years | 32.2 | 28.0, 36.7 | 66.2 | 61.4, 70.6 | 36.7 | 32.2, 41.4 | 41.8 | 37.2; 46.5 | 56.7 | 52.0; 61.3 | 31.4 | 27.0; 36.1 | 82.2 | 78.4; 85.5 | 72.5 | 68.3; 76.4 | 86.0 | 82.5; 88.9 | 42.1 | 37.3; 47.0 | 63.7 | 59.1; 68.1 | 11.8 | 09.0; 15.4 |
| 4-8 years | 33.4 | 30.9, 36.0 | 70.8 | 68.1, 73.4 | 41.1 | 38.2, 44.0 | 43.0 | 40.2; 45.8 | 61.5 | 58.7; 64.3 | 34.1 | 31.4; 36.9 | 84.8 | 82.5; 86.8 | 73.2 | 70.6; 75.6 | 86.7 | 84.2; 88.8 | 43.2 | 40.3; 46.1 | 56.0 | 53.1; 58.9 | 12.4 | 10.6; 14.4 |
| 9-11 years | 32.2 | 30.5, 33.9 | 68.3 | 66.5, 70.1 | 44.7 | 42.8, 46.6 | 45.4 | 43.5; 47.3 | 66.2 | 64.3; 68.0 | 35.6 | 33.8; 37.4 | 87.6 | 86.2; 88.8 | 74.8 | 73.2; 76.4 | 88.0 | 86.5; 89.3 | 45.3 | 43.4; 47.2 | 56.1 | 54.2; 58.0 | 13.8 | 12.5; 15.1 |
| ≥ 12 years | 34.3 | 32.3, 36.4 | 73.2 | 71.0, 75.3 | 56.1 | 53.7, 58.4 | 51.0 | 48.6; 53.4 | 72.7 | 70.4; 74.8 | 37.9 | 35.7; 40.1 | 85.8 | 83.9; 87.5 | 73.1 | 71.0; 75.0 | 91.3 | 89.7; 92.6 | 45.3 | 43.0; 47.7 | 54.7 | 52.3; 57.0 | 20.7 | 19.0; 22.5 |

VIGITEL, Surveillance of Risk and Protective Factors for Chronic Diseases through Telephone Interviews.
[a] Food consumption the day before the interview.

**Additional file 3:** Prevalence of consumption of ultra-processed foods in Brazil, by years of education, sex, and skin color/race, VIGITEL 2019.

| Subgroups | Ultra-processed foods[a] Food category | | | | | | | | | | | | | | | | | | | | | | | | | |
|---|---|---|---|---|---|---|---|---|---|---|---|---|---|---|---|---|---|---|---|---|---|---|---|---|---|---|
| Years of education, % | Soft drink | | Artificial juices | | Powdered drink mixes | | Powdered chocolate milk mix | | Flavored yogurt | | Salty snacks | | Sweet cookies and cakes | | Sweet desserts | | Reconstituted meat products | | Bread | | Sauces | | Margarine | | Ready-to-heat products | |
| | % | 95% CI | % | 95% CI | % | 95% CI | % | 95% CI | % | 95% CI | % | 95% CI | % | 95% CI | % | 95% CI | % | 95% CI | % | 95% CI | % | 95% CI | % | 95% CI | % | 95% CI |
| Total Sample | | | | | | | | | | | | | | | | | | | | | | | | | | |
| Total | 27.7 | 26.8; 28.6 | 15.0 | 14.3; 15.8 | 12.8 | 12.0; 13.5 | 11.9 | 11.2; 12.6 | 15.6 | 14.9; 16.3 | 23.9 | 23.1; 24.7 | 21.3 | 20.4; 22.1 | 25.6 | 24.8; 26.5 | 26.5 | 25.7; 27.4 | 32.8 | 31.9; 33.7 | 16.9 | 16.1; 17.6 | 42.6 | 41.7; 43.6 | 06.6 | 06.1; 07.2 |
| 0-3 years | 18.7 | 15.7; 22.1 | 12.3 | 09.8; 15.3 | 13.7 | 11.3; 16.6 | 05.6 | 04.2; 07.5 | 10.0 | 08.3; 12.0 | 25.8 | 22.7; 29.2 | 16.6 | 14.4; 19.2 | 13.7 | 11.1; 16.7 | 19.4 | 16.4; 22.9 | 19.0 | 16.3; 22.0 | 09.3 | 07.2; 11.8 | 36.8 | 33.5; 40.3 | 06.3 | 04.7; 08.3 |
| 4-8 years | 24.2 | 22.3; 26.3 | 14.3 | 12.7; 16.0 | 16.5 | 14.7; 18.4 | 09.3 | 07.9; 10.9 | 14.6 | 13.1; 16.2 | 27.3 | 25.3; 29.3 | 20.8 | 19.0; 22.7 | 18.3 | 16.6; 20.1 | 23.8 | 22.0; 25.8 | 26.0 | 24.1; 27.9 | 12.5 | 11.1; 14.1 | 47.7 | 45.6; 49.8 | 07.7 | 06.5; 09.2 |
| 9-11 years | 30.7 | 29.3; 32.2 | 17.1 | 15.9; 18.3 | 15.2 | 13.9; 16.5 | 12.9 | 11.8; 14.1 | 15.1 | 14.0; 16.1 | 25.3 | 24.1; 26.7 | 24.3 | 23.0; 25.6 | 24.9 | 23.6; 26.3 | 29.3 | 27.9; 30.7 | 34.0 | 32.6; 35.4 | 19.0 | 17.8; 20.2 | 48.0 | 46.5; 49.5 | 07.4 | 06.6; 08.4 |
| ≥ 12 years | 28.3 | 26.8; 29.9 | 13.6 | 12.4; 14.9 | 07.2 | 06.3; 08.1 | 13.7 | 12.5; 14.9 | 17.9 | 16.7; 19.3 | 19.6 | 18.3; 20.9 | 19.0 | 17.7; 20.4 | 33.8 | 32.2; 35.4 | 26.5 | 25.1; 27.9 | 38.7 | 37.1; 40.4 | 18.8 | 17.6; 20.1 | 34.1 | 32.5; 35.7 | 04.9 | 04.2; 05.9 |
| Men | | | | | | | | | | | | | | | | | | | | | | | | | | |
| Total | 33.0 | 31.5; 34.5 | 16.9 | 15.7; 18.2 | 14.9 | 13.7; 16.2 | 13.5 | 12.4; 14.7 | 14.2 | 13.1; 15.3 | 24.6 | 23.3; 26.0 | 24.0 | 22.7; 25.4 | 25.1 | 23.7; 26.5 | 31.6 | 30.2; 33.1 | 35.5 | 34.0; 37.0 | 20.3 | 19.1; 21.6 | 42.1 | 40.6; 43.7 | 07.8 | 06.9; 08.8 |
| 0-3 years | 25.1 | 19.7; 31.5 | 14.0 | 09.8; 19.6 | 13.2 | 09.5; 18.1 | 04.7 | 02.8; 07.7 | 06.7 | 04.5; 09.8 | 26.3 | 21.0; 32.4 | 18.4 | 14.6; 23.1 | 15.7 | 11.2; 21.7 | 23.9 | 18.6; 30.2 | 20.5 | 16.1; 25.7 | 09.8 | 06.9; 13.8 | 35.4 | 29.9; 41.3 | 07.2 | 04.9; 10.5 |
| 4-8 years | 29.1 | 25.9; 32.6 | 16.4 | 13.7; 19.6 | 20.5 | 17.4; 23.9 | 11.3 | 08.8; 14.4 | 12.6 | 10.4; 15.3 | 28.4 | 25.2; 32.0 | 24.1 | 21.0; 27.4 | 17.7 | 15.0; 20.8 | 28.4 | 25.2; 31.8 | 27.4 | 24.3; 30.8 | 15.1 | 12.6; 18.0 | 45.1 | 41.6; 48.7 | 09.4 | 07.3; 12.1 |
| 9-11 years | 36.0 | 33.7; 38.3 | 19.6 | 17.7; 21.6 | 17.6 | 15.6; 19.7 | 14.4 | 12.7; 16.3 | 13.5 | 12.1; 15.2 | 26.3 | 24.3; 28.5 | 27.2 | 25.1; 29.3 | 24.5 | 22.5; 26.7 | 35.1 | 32.8; 37.4 | 36.5 | 34.3; 38.7 | 23.2 | 21.2; 25.3 | 47.8 | 45.5; 50.2 | 09.0 | 07.6; 10.6 |
| ≥ 12 years | 33.5 | 31.0; 36.2 | 14.5 | 12.7; 16.5 | 07.8 | 06.5; 09.4 | 15.7 | 13.7; 17.8 | 17.6 | 15.6; 19.8 | 19.2 | 17.3; 21.3 | 21.0 | 18.8; 23.5 | 32.9 | 30.5; 35.5 | 31.1 | 28.7; 33.6 | 43.1 | 40.4; 45.8 | 22.5 | 20.5; 24.7 | 33.9 | 31.3; 36.6 | 05.1 | 03.9; 06.7 |
| Women | | | | | | | | | | | | | | | | | | | | | | | | | | |
| Total | 23.2 | 22.2; 24.3 | 13.3 | 12.5; 14.3 | 10.9 | 10.0; 11.8 | 10.5 | 09.7; 11.3 | 16.8 | 15.9; 17.7 | 23.3 | 22.3; 24.4 | 19.0 | 18.0; 19.9 | 26.1 | 25.1; 27.2 | 22.1 | 21.2; 23.2 | 30.5 | 29.4; 31.6 | 13.9 | 13.1; 14.8 | 43.1 | 41.9; 44.3 | 05.6 | 05.0; 06.3 |
| 0-3 years | 13.7 | 10.9; 17.1 | 11.0 | 08.2; 14.4 | 14.1 | 11.1; 17.7 | 06.3 | 04.4; 09.1 | 12.6 | 10.3; 15.3 | 25.3 | 21.8; 29.2 | 15.2 | 12.6; 18.3 | 12.1 | 09.6; 15.1 | 16.0 | 12.8; 19.7 | 17.8 | 14.7; 21.4 | 08.8 | 06.3; 12.3 | 37.9 | 33.9; 42.1 | 05.5 | 03.6; 08.2 |
| 4-8 years | 20.0 | 17.9; 22.2 | 12.4 | 10.8; 14.2 | 13.0 | 11.1; 15.1 | 07.5 | 06.1; 09.1 | 16.3 | 14.5; 18.2 | 26.2 | 24.1; 28.5 | 17.9 | 16.1; 19.9 | 18.8 | 16.8; 21.0 | 19.9 | 17.8; 22.1 | 24.7 | 22.7; 26.8 | 10.3 | 08.8; 12.0 | 50.0 | 47.5; 52.4 | 06.2 | 04.9; 07.7 |
| 9-11 years | 25.9 | 24.2; 27.7 | 14.8 | 13.3; 16.3 | 13.0 | 11.5; 14.7 | 11.5 | 10.2; 13.0 | 16.5 | 15.1; 17.9 | 24.4 | 22.8; 26.1 | 21.6 | 20.0; 23.3 | 25.3 | 23.7; 27.0 | 23.9 | 22.3; 25.6 | 31.7 | 30.0; 33.5 | 15.1 | 13.8; 16.6 | 48.1 | 46.2; 50.0 | 06.0 | 05.1; 07.1 |
| ≥ 12 years | 24.2 | 22.4; 26.1 | 12.9 | 11.3; 14.6 | 06.7 | 05.6; 07.9 | 12.1 | 10.8; 13.6 | 18.2 | 16.7; 19.9 | 19.9 | 18.2; 21.7 | 17.5 | 15.9; 19.1 | 34.4 | 32.5; 36.5 | 22.9 | 21.2; 24.7 | 35.4 | 33.4; 37.3 | 15.9 | 14.4; 17.5 | 34.2 | 32.2; 36.2 | 04.8 | 03.8; 06.0 |
| White | | | | | | | | | | | | | | | | | | | | | | | | | | |
| Total | 26.6 | 25.3; 28.0 | 14.0 | 12.9; 15.2 | 11.2 | 10.1; 12.3 | 11.9 | 10.9; 13.0 | 17.3 | 16.2; 18.5 | 21.1 | 19.9; 22.4 | 19.4 | 18.2; 20.6 | 29.3 | 27.9; 30.7 | 26.1 | 24.8; 27.5 | 34.9 | 33.5; 36.3 | 16.9 | 15.8; 18.1 | 37.5 | 36.0; 39.0 | 05.5 | 04.7; 06.4 |
| 0-3 years | 16.1 | 12.8; 20.2 | 07.1 | 05.1; 09.7 | 14.8 | 10.8; 19.9 | 04.5 | 02.5; 07.8 | 11.0 | 08.1; 14.7 | 24.7 | 20.0; 30.1 | 17.0 | 13.5; 21.2 | 14.2 | 10.4; 18.9 | 20.2 | 16.1; 25.0 | 20.4 | 15.9; 25.6 | 08.3 | 05.6; 12.3 | 36.1 | 30.9; 41.6 | 04.9 | 02.9; 08.0 |
| 4-8 years | 22.5 | 19.7; 25.6 | 14.1 | 11.6; 17.0 | 14.4 | 12.0; 17.2 | 09.9 | 07.7; 12.5 | 17.7 | 15.1; 20.6 | 25.0 | 22.2; 28.1 | 18.7 | 16.2; 21.6 | 21.0 | 18.3; 24.1 | 22.9 | 20.1; 26.0 | 26.7 | 23.9; 29.7 | 12.0 | 09.8; 14.6 | 46.0 | 42.7; 49.4 | 07.4 | 05.5; 09.9 |
| 9-11 years | 30.0 | 27.6; 32.5 | 15.4 | 13.5; 17.4 | 14.5 | 12.5; 16.8 | 13.0 | 11.4; 14.9 | 16.7 | 14.9; 18.7 | 21.2 | 19.3; 23.3 | 21.3 | 19.4; 23.4 | 26.7 | 24.5; 29.0 | 29.0 | 26.6; 31.4 | 34.0 | 31.7; 36.4 | 20.1 | 18.1; 22.4 | 44.1 | 41.6; 46.7 | 06.2 | 05.1; 07.6 |
| ≥ 12 years | 27.3 | 25.2; 29.5 | 13.9 | 12.1; 15.9 | 06.7 | 05.5; 08.2 | 12.9 | 11.3; 14.6 | 18.3 | 16.5; 20.2 | 18.7 | 16.9; 20.8 | 18.4 | 16.5; 20.6 | 36.8 | 34.5; 39.2 | 26.2 | 24.2; 28.3 | 41.1 | 38.7; 43.4 | 17.8 | 16.2; 19.6 | 28.8 | 26.6; 31.1 | 04.2 | 03.1; 05.7 |
| Black/Brown | | | | | | | | | | | | | | | | | | | | | | | | | | |
| Total | 28.9 | 27.7; 30.2 | 15.9 | 14.9; 17.0 | 14.0 | 13.0; 15.1 | 12.0 | 11.0; 13.0 | 14.5 | 13.7; 15.4 | 25.8 | 24.6; 26.9 | 22.9 | 21.8; 24.0 | 23.5 | 22.4; 24.6 | 27.1 | 25.9; 28.3 | 31.5 | 30.4; 32.8 | 16.9 | 15.9; 17.9 | 46.5 | 45.2; 47.8 | 07.4 | 06.6; 08.2 |
| 0-3 years | 21.0 | 16.6; 26.3 | 14.3 | 10.8; 18.6 | 13.8 | 10.5; 17.8 | 06.1 | 04.2; 08.8 | 09.2 | 07.2; 11.8 | 26.3 | 22.0; 31.1 | 16.3 | 13.2; 19.9 | 13.7 | 10.2; 18.1 | 19.4 | 15.1; 24.6 | 18.2 | 14.8; 22.3 | 10.0 | 07.2; 13.8 | 36.3 | 31.8; 41.0 | 06.5 | 04.4; 09.4 |
| 4-8 years | 25.5 | 22.8; 28.3 | 14.9 | 12.8; 17.3 | 17.8 | 15.3; 20.6 | 08.9 | 07.0; 11.2 | 13.1 | 11.3; 15.2 | 28.8 | 26.1; 31.6 | 22.6 | 20.1; 25.2 | 17.2 | 14.9; 19.7 | 25.4 | 22.7; 28.2 | 25.7 | 23.2; 28.3 | 12.9 | 11.0; 15.1 | 49.4 | 46.5; 52.3 | 07.6 | 06.0; 09.6 |
| 9-11 years | 31.6 | 29.7; 33.5 | 18.3 | 16.8; 20.0 | 15.7 | 14.1; 17.4 | 12.8 | 11.3; 14.3 | 14.4 | 13.2; 15.8 | 27.2 | 25.5; 29.0 | 25.8 | 24.1; 27.6 | 24.2 | 22.6; 26.0 | 29.5 | 27.7; 31.4 | 34.1 | 32.4; 36.0 | 18.4 | 16.9; 20.0 | 50.1 | 48.2; 52.1 | 08.3 | 07.1; 09.6 |
| ≥ 12 years | 29.7 | 27.5; 32.1 | 13.2 | 11.7; 15.0 | 07.9 | 06.6; 09.3 | 14.9 | 13.2; 16.9 | 17.4 | 15.7; 19.2 | 20.5 | 18.8; 22.4 | 19.9 | 18.2; 21.8 | 30.4 | 28.3; 32.6 | 26.5 | 24.5; 28.7 | 35.9 | 33.7; 38.2 | 19.8 | 18.0; 21.8 | 40.5 | 38.1; 42.9 | 05.9 | 04.8; 07.1 |

VIGITEL, Surveillance of Risk and Protective Factors for Chronic Diseases through Telephone Interviews.

[a]Food consumption the day before the interview.
